# Socio-Demographic and Clinical Characteristics of Psychiatric Patients Who Have Committed Suicide: Analysis of Bulgarian Regional Suicidal Registry for 10 Years

**DOI:** 10.3389/fpsyt.2021.665154

**Published:** 2021-08-19

**Authors:** Kaloyan Stoychev, Emilia Dimitrova, Vladimir Nakov, Maya Stoimenova-Popova, Petranka Chumpalova, Ivanka Veleva, Eleonora Mineva-Dimitrova, Dancho Dekov

**Affiliations:** ^1^Department of Psychiatry and Medical Psychology, Medical University Pleven, Pleven, Bulgaria; ^2^Department of Psychiatry, ‘Dr. Georgi Stranski' University Hospital, Pleven, Bulgaria; ^3^Department of Mental Health, National Center of Public Health and Analyses, Sofia, Bulgaria; ^4^Department of Public Health Sciences, Medical University Pleven, Pleven, Bulgaria; ^5^Deparment of General Medicine, Forensic Medicine, and Deontology, Medical University Pleven, Pleven, Bulgaria

**Keywords:** suicide, risk factors, study, Bulgaria, mental disorders

## Abstract

**Introduction:** Suicide is a major public health problem but factors determining suicide risk are still unclear. Studies in this field in Bulgaria are limited, especially on a regional level.

**Methods:** By a cross-sectional design, we accessed the medical records of all psychiatric patients committed suicide over a 10-year period (2009–2018) in one major administrative region of Bulgaria. A statistical analysis was performed of the association between age of suicide as an indirect yet measurable expression of the underlying suicidal diathesis and a number of socio-demographic and clinical characteristics.

**Results:** Seventy-seven of 281 suicides (28%) had psychiatric records. Most common diagnoses were mood disorders (44%), followed by schizophrenia (27%), anxiety disorders (10%), substance use disorders (9%) and organic conditions (8%). Male gender, single/divorced marital status, early illness onset, co-occurring substance misuse and lower educational attainment (for patients aged below 70) were significantly associated with earlier age of suicide whereas past suicide attempts and psychiatric hospitalizations, comorbid somatic conditions and unemployment showed insignificant association. Substantial proportion of patients (60%) had contacted psychiatric service in the year preceding suicide, with nearly half of these encounters being within 30 days of the accident.

**Conclusion:** Severe mental disorders are major suicide risk factor with additional contribution of certain socio-demographic and illness-related characteristics. Monitoring for suicidality must be constant in chronic psychiatric patients. Registration of suicide cases in Bulgaria needs improvement in terms of information concerning mental health. More studies with larger samples and longitudinal design are needed to further elucidate distal and proximal suicide risk factors.

## Background

Suicide is a global health burden and an indicator for the mental health of populations and societies (1). In Bulgaria, suicide is a major public health problem with an estimated prevalence rate from 9 to 12 per 100,000 residents between 2009 and 2013 (2). However, studies on suicide are still quite deficient compared to other European countries, and few have examined regional aspects of suicidality (3).

Consistent global data from the past several decades confirm that most victims of suicide have a mental disorder and various studies report such in as many as 90% of all cases (4). Beyond that inference, however, factors determining acute and chronic suicide risk are still poorly understood (5). Among them are some socio-demographic (6) and clinical (7–9) variables that have been sustainably associated with suicidal behavior, especially in committed suicides (6).

Against the background outlined above, the present study aimed to gather and analyze socio-demographic and clinical data of all subjects with mental disorders who have died by suicide over a 10 year period (2009–2018) in one of the 28 major administrative regions Bulgaria.

## Materials and Methods

### Data Collection Procedures, Study Design, Inventory, and Variables

Data collection was based on the procedure of suicide registration in Bulgaria which is a multi-step process. Until 2017, a responsible healthcare official (e.g., coroner, emergency medic etc.) entered information about each suicide case into two specific paper forms. A unified electronic register replaced these documents from 2017 on, including data about the demography of the victim (age, sex, residence, marital status) and the date, method, and location of the suicidal act (2). All collected data are then processed by the regional departments of the Ministry of Healthcare and sent to the National Center for Public Health and Analyses (NCPHA). After software examination allowing detection and removal of redundant information, data are finally stored there for statistical and public health policy purposes.

Adopting a retrospective design, we accessed NCPHA database and analyzed all suicide cases that occurred between January 1, 2009, and December 31, 2018, in the Pleven region - one of the 28 major administrative units in Bulgaria, with about 240,000 residents as of 2018. During the studied period, 281 cases of suicide were confirmed. All of them were checked against the electronic databases of the participating healthcare institutions—the Department of Psychiatry at the University General Hospital in Pleven and all psychiatric outpatient centers in the region. Thus, collected psychiatric information was not limited to hospitalized patients solely but captured those receiving treatment in a community setting. Seventy-seven subjects had medical records attesting to mental illness. We used a study-customized inventory including socio-demographic, and illness-related characteristics (Tables 1, 3) to extract and analyze their association with the age of the subjects who committed suicide. Age itself might be considered indicative of overall suicide risk insofar as, while suicide rate generally increases with age (10, 11), suicidal behavior occurring earlier in the life cycle is likely to reflect a stronger underlying diathesis (12). In addition to that, the time interval between the suicidal act and the last documented psychiatric contact was calculated.

**Table 1:** Socio-demographic characteristics of patients with psychiatric medical records for the period 2009–2018.

**Characteristic**	**Number (females/males) or value (years)**	**Relative prevalence %**
Gender		
- Male	48	62.3
- Female	29	37.7
Mean age		
- Male	52.67	n.a.
- Female	60.52	n.a.
Marital status		
- Married	32 (13/19)	41.6
- Divorced/single	34 (7/27)	44.2
- Widowed	11 (9/2)	14.3
Employment status		
- Employed	11 (6/5)	14.3
- Unemployed	17 (4/13)	22.1
- Retired	28 (14/14)	36.3
- Disabled	21 (5/16)	27.3
Education		
- College/University degree	7 (4/3)	9.1
- High school	56 (19/37)	72.7
- Elementary school	14 (6/8)	18.2
Lifetime clinical history		
- Past psychiatric hospitalizations	48 (16/32)	62.3
- Past hospitalizations – mean (*n*)	3.2 (2.5/3.8)	n.a.
- Past suicide attempts	20 (7/13)	26.0
- Past suicide attempts – mean (*n*)	1.5 (1.14/1.69)	n.a.
- Co-occurring SUD^a^	8 (2/6)	10.4
- Co-occurring somatic illness	40 (14/26)	51.9
- Mental illness onset—mean (years)	41.4 (45.3/39.1)	n.a.
Suicide methods		
- ***Violent methods***	67 (22/45)	87.0
a. Hanging	39 (11/28)	50.6
b. Firearms	7 (0/7)	9.1
c. Jumping from high places	17 (8/9)	22.1
d. Traffic death	3 (2/1)	3.9
e. self-immolation	1 (1/0)	1.3
- ***Non-violent methods (females/males)***	10 (7/3)	13.0
a. Drug overdose	7 (5/2)	9.1
b. Drowning	3 (2/1)	3.9

a *Substance Use Disorder (other than caffeine or nicotine)*.

### Statistical Analysis

Study results, i.e., demographic, clinical, and suicide methods characteristics, were described as prevalence rates, means, and correlation coefficients. Categorical variables were presented as numbers and percentages, while continuous variables (when normally distributed) were expressed as means ± standard deviations (SDs). Comparisons between continuous variables for each pair of groups were performed by the Student's t-test (for normally distributed variables) and the Mann-Whitney U test (for variables with skewed distribution). Comparisons among continuous variables in three or more groups were performed by means of one-way ANOVA (for normally distributed variables) and the Kruskal-Wallis H test (for variables with skewed distribution). The magnitude of the relationship between continuous variables was measured by the Pearson correlation coefficient for linear relationships. Analysis was performed using IBM SPSS v. 25.0 software running on top of Windows 10 operating system equipped with Microsoft 2016 office pack. Statistical significance was set at *p* ≤ 0.05, i.e., a 95% confidence level was established.

Data for the mid-period population was obtained from the 2011 National Population Census of Bulgaria (13) with a small adjustment based on the 2018 annual report for population and demographic processes issued by the Bulgarian National Statistical Institute (14).

### Ethical Considerations

The study was conducted per the declaration of Helsinki (15) and was approved by the Institutional Ethics Review Board of Pleven Medical University and NCPHA. The subjects' personal data were protected by pseudonymization with access restricted to the investigators only.

## Results

### Socio-Demographic Characteristics

Basic socio-demographic and clinical characteristics of the sample are presented in Table 1.

Men who accounted for nearly 2/3 of the sample were overrepresented among divorced/single, unemployed and disabled patients, those with past psychiatric hospitalizations and suicide attempts, and those with a co-occurring somatic/neurological illness. In addition to that, they had earlier onset of mental illness and were more likely to use a violent suicidal method. On the other hand, women were more often widowed and tended to have somewhat higher education as measured by the prevalence of high-school or college/university academic degrees.

### Psychiatric Diagnoses

The distribution of patients according to their psychiatric diagnoses is presented in Table 2. All diagnostic codes were based on the ICD-10 classification of diseases and related health problems, chapter V, Mental and behavioral disorders (16).

**Table 2:** Distribution of patients according to ICD-10 diagnostic code.

**Diagnostic category**	**Number of cases and sex (f/m)**	**Gender (females/males)**	**Relative prevalence (%)**
**F00-F09 organic, including symptomatic, mental disorders:**	6	2/4	7.8
- Organic delusional disorder	1	0/1	
- Organic mood disorder	1	1/0	
- Organic anxiety disorder	1	0/1	
- Other specified mental disorder due to known physiological condition	1	1/0	
- Organic personality disorder	2	0/2	
**F10-F19 Mental and behavioral disorders due to psychoactive substance use:**	7	2/5	9.1
- Alcohol dependence	6	1/5	
- Opioid dependence	1	1/0	
**F20-F29 Schizophrenia, schizotypal and delusional disorders:**	21	3/18	27.3
- Schizophrenia, paranoid type	20	3/17	
- Schizoaffective disorder	1	0/1	
**F30-F39 Mood (affective) disorders:**	34	19/15	44.2
- Single depressive episode	4	2/2	
- Recurrent depressive disorder	21	14/7	
- Bipolar disorder	9	3/6	
**F40-F49 Neurotic, stress-related and somatoform disorders:**	8	3/5	10.4
- Panic disorder	1	0/1	
- Mixed anxiety-depressive disorder	3	1/2	
- Generalized anxiety disorder	1	1/0	
- Obsessive-compulsive disorder	1	0/1	
- Adjustment disorder	1	0/1	
- Somatization disorder	1	1/0	
**F60-F69 Disorders of adult personality and behavior:**	1	0/1	1.3
- Paranoid personality disorder	1	0/1	
**Total**	77	29/48	100.0

Within the group of organic mental disorders, symptoms were accounted for by cerebrovascular disease (*n* = 3), epilepsy (*n* = 2), and Parkinson's disease (*n* = 1). Substance use disorders were mainly represented by alcohol dependence (~86% of all cases). Similarly, the psychotic disorders group (F20-F29) comprised almost exclusively paranoid schizophrenia subjects (95%). The group of mood disorders (*n* = 34) was the largest and in the group of anxiety disorders (*n* = 7) the diagnostic codes were most diverse. Nine of the patients (11.7%) had a co-occurring mental disorder–a substance-use disorder in eight of them (mainly alcohol and cannabis and amphetamines use disorder), and atypical anorexia nervosa in a woman with recurrent depression. All comorbid subjects were classified according to their chronologically and clinically primary disorder as judged by the investigators.

### Association of Demographic and Clinical Characteristics With the Age of Suicide Completion

The mean age of suicide completion for the sample was 55.62 years, significantly lower in males (52.67 years) than in females (60.52 years) (*t* = −2.071, df = 75, *p* = 0.042)-Table 3. Divorced or single patients demonstrated significantly lower mean age (45.97 years) compared to married/cohabiting ones (60.41 years) [*F*_(2, 74)_ =17.682, *p* < 0.001]. In comparison, occupational status did not affect the age of suicide as both employed and unemployed subjects had a very similar mean age of suicide - 41.72 and 41.88 years, respectively (*t* = −0.035, df = 19.234, *p* > 0.05). A significant difference was detected in the age of suicide as a function of educational level [$H_{\left(2\right)}^{2}$ = 13.831, *N* = 77, *p* < 0.01]. It was at the expense of high school (mean age 51.91 years, mean rank 33.87) and elementary school (mean age 70.36 years, mean rank 58.68) educational levels (*U* = 3.713, *p* < 0.01)—Table 3.

**Table 3:** Comparison of mean age of suicide corresponding to selected socio-demographic and clinical characteristics.

**Characteristic**	**Mean age**	**Statistical significance (*p* < 0.05)**
Gender		
- Females	60.52	n.s.
- Males	52.67	*p* = 0.042
Marital status		
- Married	60.41	n.s.
- Divorced/single	45.97	*p* < 0.001
Employment status		
- Employed	41.72	n.s.
- Unemployed	41.88	n.s.
Education		
- College/University degree	55.25	n.s.
- High school	51.91	*p* < 0.01
- Elementary school	70.36	*p* < 0.01
Past suicide attempt		
- With	54.90	n.s.
- Without	56.65	n.s.
Past psychiatric hospitalization		
- With	54.85	n.s.
- Without	56.86	n.s.
Co-occurring SUD		
- With	44.38	n.s.^*^
- Without	56.91	n.s.
- Schizophrenia with SUD	43.80	n.s.
- Schizophrenia without SUD	44.88	n.s.
Co-occurring somatic illness		
- With	60.47	n.s.
- Without	48.71	*p* = 0.002

* *r_pb_r = −0,234, p = 0.040, N = 77*.

As regards the diagnostic group consisting of >1 case (Table 4), schizophrenia demonstrated the lowest mean age of suicide (44.62 years), and organic disorders unsurprisingly had the highest (66.83 years). No statistical age difference was found between male and female patients within each diagnostic category, though in most of them, male suicides were younger. Of note, age of mental illness onset (41.4 years, Table 1) showed a positive correlation with age of suicide completion (*r* = 0.754, *p* < 0.001, *N* = 77), suggesting a younger age of suicide in patients with an earlier occurrence of psychiatric disorder.

**Table 4:** Mean age of suicide completion for the sample in total and according to patients' diagnostic group with corresponding values for male and female subjects.

**Diagnostic group**	**Sample (*n* = 77)**	**Females (*n* = 29)**	**Males (*n* = 48)**	**Statistical significance (*p* < 0.05)**
F00-F09	66.83 ± 12.54	67.50 ± 19.09	66.50 ± 11.85	n.s.
F10-F19	55.57 ± 20.00	29.50 ± 9.19	66 ± 10.15	n.s.
F20-F29	44.62 ± 10.20	41.00 ± 14.00	45.22 ± 9.83	n.s.
F30-F39	61.35 ± 15.13	65.42 ± 14.00	56.60 ± 15.52	n.s.
F40-F49	53.50 ± 20.27	65.00 ± 19.29	46.60 ± 19.35	n.s

History of previous suicide attempt(s)s was not associated with a significant difference in the mean age of suicide (54.90 years, mean rank 38.45) compared to that of patients without preceding attempt(s) (56.90 years, mean rank 39.19 years) (*U* = 559.000, *N* = 77, *p* > 0.05). In the same way, past psychiatric hospitalizations did not substantially impact the age of suicide, which was 54.85 years for those with former inpatient treatment vs. 56.86 years for those without (*U* = 655.000, *N* = 77, *p* = 0.666). Interestingly, the slight age difference between the two groups seemed to be driven exclusively by female subjects with 59.19 years mean age for previously hospitalized vs. 62.15 years for non-hospitalized patients. In contrast, in males, such a trend was not present (52.69 vs. 52.56 years.). Still, the difference between hospitalized (mean rank 14.50, *N* = 16) and non-hospitalized (mean rank 15.62, *n* = 13) women were not at the level of statistical significance (*U* = 96.000, *N* = 29, *p* = 0.746).

Notably, in subjects with both mental and substance use disorder, a weak correlational association was found (Table 3, bottom), i.e., substance misuse appeared to influence the age of suicide, accounting for about 5.5% of its variation. Supporting such an assumption, the mean age of dually diagnosed subjects (mean rank 24.36, *n* = 8) was distinctly lower (44.38 years) compared with the single disorder group (56.91 years) (mean rank 40.70, *N* = 69). However, the difference did not reach statistical significance (*U* = 159.000, *N* = 77, *p* > 0.05). As five of the dually diagnosed subjects (i.e., >60%) had schizophrenia, their mean age of suicide (43.80 years) was compared to that of non-comorbid schizophrenics (44.88 years) again without significant difference being outlined (*t* = 0.202, df = 19, *p* = 0.842).

The impact of co-occurring somatic illness on suicide risk was evaluated by comparing the mean age of suicide committed by somatically ill patients and somatically healthy ones. Patients with organic mental disorders were excluded from the analysis because their somatic condition was considered primary, i.e., apparently contributing to suicide risk. Of the remaining 71 patients, 36 (>50%) had at least one somatic illness, with arterial hypertension and non-insulin-dependent diabetes being the most frequent diagnoses. Surprisingly, somatically healthy subjects were significantly younger at the time of suicide (48.71 years) compared to those with somatic illness (60.47 years) (*t* = −3.199, df = 69, *p* = 0.002).

### Time Between Suicide and Last Contact With a Mental Health Care Provider

We looked at the time interval between suicide and last documented psychiatric contact for the two groups of individuals in our sample—those treated as outpatients only and those with psychiatric hospitalization(s). There was a differentiation albeit not statistically significant (*U* = 799.000, *N* = 77, *p* = 0.279)-with the mean value for the total sample of 1,172.47 days the time between suicide and the last psychiatric contact was shorter in the ambulatory cases (1,025.90 days) than those treated in hospital settings before the suicide act (1,261.02 days). Of 29 ambulatory patients, 21 had seen a psychiatrist within 12 months of suicide (10 in the last 30 days). For patients whose last contact was hospitalization (*n* = 48), it was in the year preceding suicide in 25 of the cases, and in 11, the hospital discharge was within 30 days of suicide.

## Discussion

The present study explored socio-demographic and clinical characteristics of patients with a medical record for mental disorder sampled among all suicide cases that occurred in the major Bulgarian administrative region of Pleven over 10 years (2009–2018).

**Figure 1:**
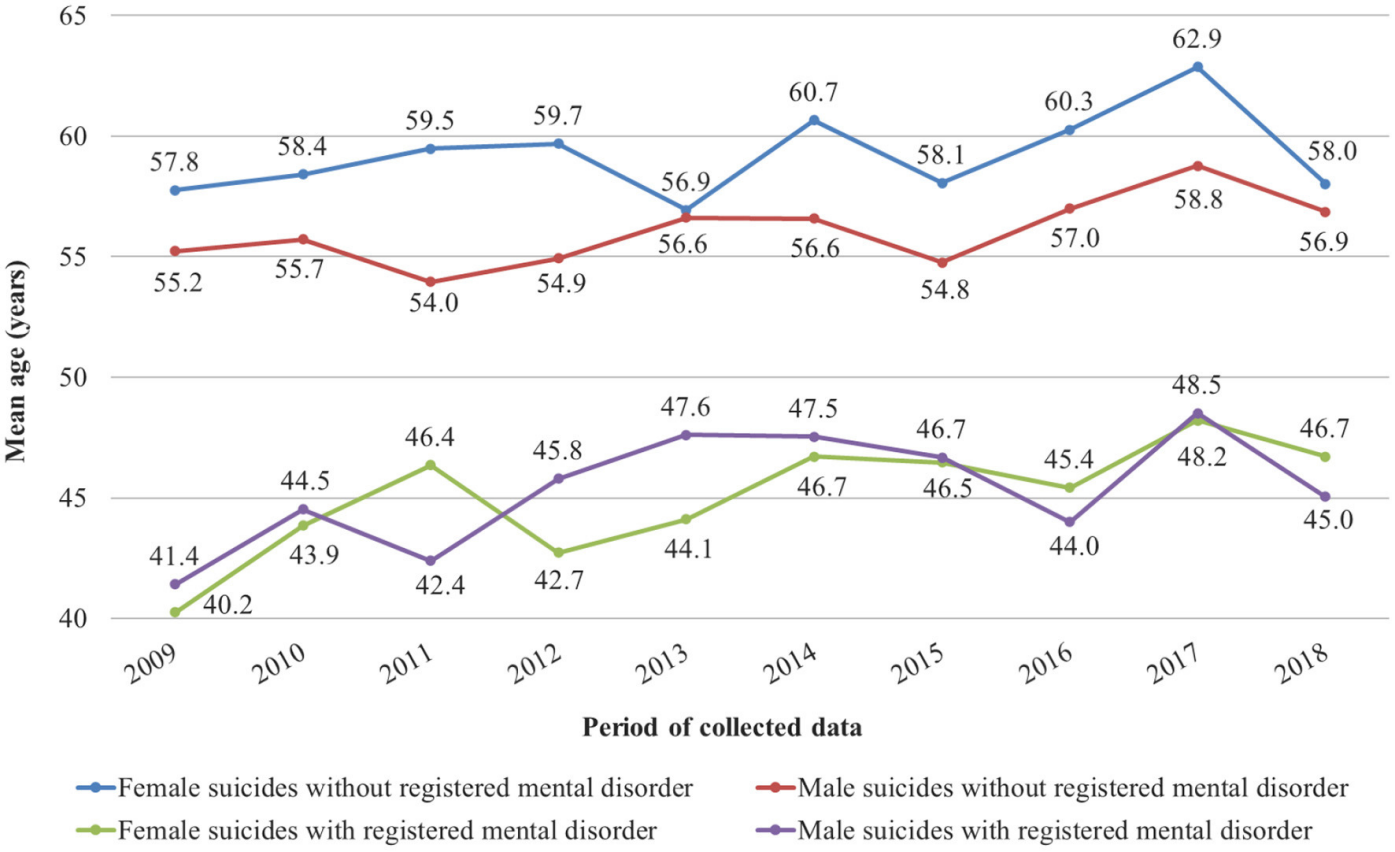
Dynamics of the mean age of suicide completion for subjects with and without a registered mental disorder in Bulgaria (2009–2018).

From a clinical perspective, in line with findings from studies in Europe (17), China (18), and elsewhere (12), the most common psychiatric diagnoses among suicide victims were mood disorders (44%) and particularly major depression (33%), followed by schizophrenia (27%), anxiety, somatoform and stress-related disorders (10%), substance use disorders (9%), and organic mental disorders (8%). Hence, it could be presumed that chronic and severe mental illnesses are a factor magnifying suicide risk, expressed in an earlier age of suicide. Reinforcing such an assumption, analysis of the Bulgarian National Center for Public Health (NCPHA) database for the same period as the one in our study reveals a discernibly younger age of suicide of patients with a registered mental disorder compared to those without (19)—Figure 1.

In agreement with literature data showing twice more common suicides in men (20), our study found that more than 60% of the suicide victims were males. Besides, male suicides were significantly younger than females. This finding is at odds with other studies focusing on similar samples in Southeast Europe (21) and may be associated with several factors. First, the mean age of suicide completion in Bulgaria is generally lower among men [(22), Figure 1], and this trend might have been reflected in our sample. Second, male patients had an earlier age of mental illness onset (39.1 vs. 45.3 years for females—Table 1), which could also explain the younger age of male suicides given the positive chronological correlation we found between the onset of psychiatric symptoms and the manifestation of suicidal behavior. Finally, the relative prevalence of schizophrenia, a disorder generally associated with a higher rate of suicide among younger age groups (23), was much higher in males (37.5% of all diagnoses) than in females (10.3%). Schizophrenic men also had a considerably higher rate of past suicide attempts, with 7 of them having more than one attempt. This rate resulted in a higher mean number of previous attempts among men, which is in disparity with literature data ascribing a higher incidence of non-fatal suicidal acts to women (24).

Another important finding was the higher age of suicide in married patients vs. divorced or single ones - 61.41 vs. 45.97 years, respectively. This result, implying a protective impact of marriage on suicidal behavior, agrees with evidence from many extensive population-based studies (25–27). However, considering the presence of at least one study propounding higher suicide risk in married psychiatric patients (28), we also compared the rate of past suicide attempts in married vs. divorced or single patients. Again, marriage seemed to act protectively on suicidal behavior. Only 30% (6 out of 26) of patients with a partner had had prior suicide attempts compared to 60% (12 out of 22) in the single/divorced group.

Contrary to the extensively advocated in the literature (29), we did not identify an association between unemployment and suicidal risk—both employed and unemployed persons had similar mean ages of suicide: 41.72 and 41.88 years, respectively. This finding may be explained by the gradual decline of the unemployment-associated suicidal risk over time, as reported by some studies (30). In this model, the increased suicidal risk may be present in the first several years following job loss, but it fades away after that. Therefore, if unemployed subjects in our sample had been in this position for a long time, the interval of heightened risk had already expired, and other risk factors comparable with those working in employed patients may have driven the suicidal behavior.

In our study, high school graduates, which represented >70% of the sample, had the youngest age of suicide (51.91 years), followed by college/university degree holders (55.25 years) and elementary school attainers (70.36 years). The latter group might be considered a conveyor of lower suicide risk expressed by its highest mean age of suicide. This finding agrees with data reported by other studies in Europe showing that suicide victims significantly more frequently have higher educational attainment (31). However, the validity of such an assumption is limited by the fact that in Bulgaria, for reasons concerning historical and socio-economic development, people aged 70+ years generally have had poorer access to education compared to the subsequent generations. That being said, a collation between high-school and college/university degree graduates only reveals discernibly younger age for the former (51.91 years) compared to the latter (55.25 years). This fact supports data from other European population-based studies, showing that lower educational attainment might be a risk factor for suicide (17, 32).

Another intriguing finding is the higher age of suicide of somatically ill psychiatric patients than those without a co-occurring medical condition. Such a result seems contra-intuitive since the presence of somatic illnesses has been reported as independent risk factors for suicide (17, 33). Hence, earlier occurrence of suicidal behavior among somatically ill psychiatric patients should be expected. This controversy may be explained in two ways. First, psychiatric records in Bulgaria only state the presence of somatic condition without marking its age of onset. This warrants the assumption that most of the somatic illnesses affecting patients might have started later in life - months to few years before suicide—thereby “not having enough time” to exert its incremental effect on suicide peril. Second, it may be related to dissimilarity in mental disorder severity between somatically healthy and somatically ill patients. As stated by some studies (34–36), the effect of mental disorders on suicidal risk is mediated mainly by a general psychopathology risk factor, not bound to a specific diagnosis. Hence, patients without comorbid somatic conditions may all have been carriers of higher general psychopathology with corresponding earlier set off of suicidal behavior that explains the lower suicide age. Such an assumption is supported by at least one recent prospective study, in which the magnitude of the general psychopathology factor effect on suicide attempts has been significantly higher in younger than in older adults (37).

The average interval between last mental health service (MHS) contact and suicide was 1,172.47 days, distinctively shorter for ambulatory treated subjects (1,026 days) compared to hospital-discharged (1,261 days). In the last 12 months before suicide, 59.7% of the patients had seen a psychiatrist. In addition to that, 27% had sought such a contact within the month preceding the fatal accident. While the rate of contact with MHS in the 12-months before suicide in our study (59.7%) is much higher than the average 31% reported in the literature (38), a possible explanation of this is the high proportion of patients treated on an outpatient basis who have easier access to mental healthcare services in Bulgaria. The latter may also elucidate the shorter mean period between last contact and suicide among them.

In only 77 out of the 281 subjects (i.e., ~28%) who committed suicide between 2009 and 2018 in the Pleven region, there were medical records confirming the mental disorder. This proportion, which is in contrast with global epidemiological data (12), could be interpreted in several ways. On the one hand, it is possible that the prevalence of mental disorders among suicides in that particular Bulgarian region for the specified period might have really been much lower than averagely reported. Supporting such a conjecture, a recent broad-scope review (39) concludes that the incidence of mental disorders among suicide victims does show a substantial geographically-driven variation and is overall lower in developing countries. On the other hand, it is also possible that the lower prevalence is a consequence of self-restrained help-seeking behavior in patients who otherwise experience psychiatric symptoms. According to some studies, such a pattern is typical for societies with a high prevalence of mental illness-associated stigmatization (39). However, it is much more likely that the finding is a result of methodological shortcomings. First, the low rate we found might be due to the sample size. Studies in Europe and elsewhere, utilizing similar small size samples, generally report lower prevalence of mental illness in suicide victims (30–57%)—for example Manoranjitham et al. (40), Innamorati et al. (41), Graham and Burvill (42), Sun and Jia (43). Second, in Bulgaria, electronic databases of primary care and mental health care providers are not connected. For this reason, a substantial amount of data referring to the primary care treated mental conditions remains hidden for specialists.

The main advantage of our study is its comprehensive design allowing access to the records of all psychiatric patients in the region and collecting data for a relatively long period. Thus, specific enduring trends could be outlined, making findings generalizable to a larger populational level. Tha major limitations are the cross-sectional design and the small sample size, which substantially restrict the validity of the associations recognized. Another flaw is the lack of individual exploration of each suicide case (“psychological autopsy”) which is the most reliable tool in studying the relationships between various factors contributing to suicide (44). In this respect, collecting information for psychosocial dysfunction immediately preceding suicide should be implemented in the suicide registration process in Bulgaria.

## Conclusion

Our study confirms that severe and chronic mental disorders constitute a significant risk factor for suicide, especially if symptoms have started early in life and are complicated by a co-occurring substance use disorder. From a socio-demographic perspective, factors associated with earlier suicide (i.e., higher suicide risk) are male gender, divorced or single marital status, and lower educational attainment (for subjects aged below 70 years). A significant proportion of patients who committed suicide have contacted psychiatric services in the year preceding the accident, making regular and focused screening for suicidal ideation imperative. Future longitudinal studies with larger samples assembling both primary and mental health care treated patients and employing individual exploration of each suicide are needed to verify or dispute these presumptions.

## Data Availability Statement

The data analyzed in this study is subject to the following licenses/restrictions: GDPR. Requests to access these datasets should be directed to v.nakov@ncpha.government.

## Ethics Statement

The studies involving human participants were reviewed and approved by Medical University Pleven—Ethics Committee on scientific research. Written informed consent for participation was not required for this study in accordance with the national legislation and the institutional requirements.

## Author Contributions

KS, VN, and ED: data collection, analysis, and text preparation. DD, IV, PC, and MS-P: data collection. EM-D: statistical analysis. All authors contributed to the article and approved the submitted version.

## Conflict of Interest

The authors declare that the research was conducted in the absence of any commercial or financial relationships that could be construed as a potential conflict of interest.

## Publisher's Note

All claims expressed in this article are solely those of the authors and do not necessarily represent those of their affiliated organizations, or those of the publisher, the editors and the reviewers. Any product that may be evaluated in this article, or claim that may be made by its manufacturer, is not guaranteed or endorsed by the publisher.
