# Corrigendum: Socio-Demographic and Clinical Characteristics of Psychiatric Patients Who Have Committed Suicide: Analysis of Bulgarian Regional Suicidal Registry for 10 Years

**DOI:** 10.3389/fpsyt.2021.812498

**Published:** 2021-12-06

**Authors:** Kaloyan Stoychev, Emilia Dimitrova, Vladimir Nakov, Maya Stoimenova-Popova, Petranka Chumpalova, Ivanka Veleva, Eleonora Mineva-Dimitrova, Dancho Dekov

**Affiliations:** ^1^Department of Psychiatry and Medical Psychology, Medical University Pleven, Pleven, Bulgaria; ^2^Department of Psychiatry, ‘Dr. Georgi Stranski’ University Hospital, Pleven, Bulgaria; ^3^Department of Mental Health, National Center of Public Health and Analyses, Sofia, Bulgaria; ^4^Department of Public Health Sciences, Medical University Pleven, Pleven, Bulgaria; ^5^Deparment of General Medicine, Forensic Medicine, and Deontology, Medical University Pleven, Pleven, Bulgaria

**Keywords:** suicide, risk factors, study, Bulgaria, mental disorders

An author name was incorrectly spelled as Kalyan Stoychev. The correct spelling is Kaloyan Stoychev.

In the original article, there was an error. “Pearson correlation coefficient” was incorrectly written as “Person correlation coefficient.”

A correction has been made to **Materials and Methods, Statistical Analysis**, paragraph 1:

“Study results, i.e., demographic, clinical, and suicide methods characteristics, were described as prevalence rates, means, and correlation coefficients. Categorical variables were presented as numbers and percentages, while continuous variables (when normally distributed) were expressed as means ± standard deviations (SDs). Comparisons between continuous variables for each pair of groups were performed by the Student's t-test (for normally distributed variables) and the Mann-Whitney U test (for variables with skewed distribution). Comparisons among continuous variables in three or more groups were performed by means of one-way ANOVA (for normally distributed variables) and the Kruskal-Wallis H test (for variables with skewed distribution). The magnitude of the relationship between continuous variables was measured by the Pearson correlation coefficient for linear relationships. Analysis was performed using IBM SPSS v. 25.0 software running on top of Windows 10 operating system equipped with Microsoft 2016 office pack. Statistical significance was set at *p* ≤ 0.05, i.e., a 95% confidence level was established.”

The authors apologize for these errors and state that this does not change the scientific conclusions of the article in any way. The original article has been updated.

## Publisher's Note

All claims expressed in this article are solely those of the authors and do not necessarily represent those of their affiliated organizations, or those of the publisher, the editors and the reviewers. Any product that may be evaluated in this article, or claim that may be made by its manufacturer, is not guaranteed or endorsed by the publisher.

